# Chronic cigarette smoking enhances spontaneous release of tumour necrosis factor-α from alveolar macrophages of rats

**DOI:** 10.1155/S0962935193000602

**Published:** 1993

**Authors:** G. P. Pessina, L. Paulesu, F. Corradeschi, E. Luzzi, M. Tanzini, C. Aldinucci, A. Di Stefano, V. Bocci

**Affiliations:** 1 Institute of General Physiology University of Siena via Laterina 8 Siena 53100 Italy; 2 Department of Molecular Biology University of Siena Siena 53100 Italy

## Abstract

Some biological effects of chronic cigarette smoking (two cigarettes for 2 h, daily for 4 months) in rats were evaluated. During the smoking period, body weight of smoker rats was always significantly lower than that of control rats. Immediately after the last smoking session the carboxyhaemoglobin concentration in the blood was about 8.5% and the polymorphonuclear cells in the bronchoalveolar fluid increased significantly. At the same time, enzymatic analyses on the supernatants of bronchoalveolar fluid revealed a significant increase of β-glucuronidase in the smoker group. Alveolar macrophages, collected 0, 8 and 24 h after the last smoking session, significantly increased the generation of superoxide anion and, after incubation for 24 h at 37^°^ C in a humidified atmosphere, released significantly high amounts of TNF-α. When challenged with lipopolysaccharide, alveolar macrophages of smoker rats released much more TNF-α but, in such a case, TNF-α release was about one half of that observed in the control group. Peritoneal macrophages of both control and smoker rats were unable either to generate high levels of superoxide anion or to release significant amounts of TNF-α. The results clearly demonstrated the activated state of alveolar macrophages and the resting state of peritoneal macrophages.


Mediators of Inflammation 2, 423–428 (1993)

### Chronic cigarette smoking enhances spontaneous release of tumour necrosis factor-$\alpha$ from alveolar macrophages of rats

G. P. Pessina,[1] L. Paulesu,[1] F. Corradeschi,[1] E. Luzzi,[1] M. Tanzini,[1] C. Aldinucci,[1] A. Di Stefano[2] and V. Bocci[1,CA]

[1]Institute of General Physiology University of Siena, via Laterina 8, 53100 Siena, Italy; [2]Department of Molecular Biology, University of Siena, 53100 Siena, Italy

[CA] Corresponding Author

**SOME biological effects of chronic cigarette smoking (two cigarettes for 2 h, daily for 4 months) in rats were evaluated. During the smoking period, body weight of smoker rats was always significantly lower than that of control rats. Immediately after the last smoking session the carboxyhaemoglobin concentration in the blood was about 8.5% and the polymorphonuclear cells in the bronchoalveolar fluid increased significantly. At the same time, enzymatic analyses on the supernatants of bronchoalveolar fluid revealed a significant increase of $\beta$-glucuronidase in the smoker group. Alveolar macrophages, collected 0, 8 and 24 h after the last smoking session, significantly increased the generation of superoxide anion and, after incubation for 24 h at 37°C in a humidified atmosphere, released significantly high amounts of TNF-$\alpha$. When challenged with lipopolysaccharide, alveolar macrophages of smoker rats released much more TNF-$\alpha$ but, in such a case, TNF-$\alpha$ release was about one half of that observed in the control group. Peritoneal macrophages of both control and smoker rats were unable either to generate high levels of superoxide anion or to release significant amounts of TNF-$\alpha$. The results clearly demonstrated the activated state of alveolar macrophages and the resting state of peritoneal macrophages.**

**Key words:** Alveolar macrophages, Cigarette smoking, Inflammation, Interferon, Tumour necrosis factor-$\alpha$

### Introduction

The lung represents a frequent site of inflammation because of its direct exposure to noxious agents, antigenic materials and invasion by microorganisms. Cigarette smoking, exposing the surface of the lower respiratory tract to more than 4000 chemical constituents such as polynuclear aromatic hydrocarbons and tobacco specific nitrosamines,[1] has been associated with inflammatory processes.[2] The development of pulmonary inflammation is known to involve cellular events controlled by a variety of cytokines. These mediate interactions between macrophages (M$\phi$), lymphocytes and granulocytes and represent an important event in the regulation of airway inflammation.[3,4] In fact there is evidence that alveolar M$\phi$, which are derived from blood monocytes, may mediate many of the harmful effects of smoking, exerting their activity at the inflammatory sites partly directly and partly through the action of tumour necrosis factor (TNF) and other immunological mediators.[5] Moreover interactions between TNF and other cytokines are of pivotal importance in the mediation of the activation state of M$\phi$.[6] TNF-$\alpha$ is a protein with a wide range of biological and inflammatory activities. The pleiotropic nature of its action is due to the fact that TNF receptors are present on virtually all cells examined and TNF action leads to the activation of a large array of cellular genes and of multiple signal transduction pathways.[7] Moreover TNF is chemotactic for monocytes and polymorphonuclear cells (PMN), stimulates phagocytosis, adherence to endothelium and superoxide anion production by these cells, induces procoagulant activity and activates M$\phi$ resulting in interleukin-1 and prostaglandin-E2 production.[8] Thus, the locally regulated generation of TNF-$\alpha$ at the sites of injury represents an important autocrine–paracrine control mechanism operative in the inflammatory response.

Despite the wide consumption of cigarettes and the important role of alveolar M$\phi$ in the lung, little is known about the effect of long-term exposure to cigarette smoke, under controlled conditions, concerning the eventual release of cytokines. In a previous work[9] the authors demonstrated that, after a single episode of acute cigarette smoking, alveolar M$\phi$ became activated and released TNF-$\alpha$. The aim of this study was to investigate the effect of chronic smoke in the activation of alveolar M$\phi$ of rats and in the release of cytokines such as TNF-$\alpha$ and interferon (IFN). The production of superoxide anion by alveolar M$\phi$ and the eventual variations of enzymes such as $\beta$-glucuronidase and lactate dehydrogenase in both bronchoalveolar fluid (BAL)

© 1993 Rapid Communications of Oxford Ltd and peritoneal fluid (PF) were also examined. Finally we evaluated whether the release of cytokines after stimulation of alveolar and peritoneal M$\phi$ of smoker rats with lypopolysaccharide would be stimulated or depressed.

### Materials and Methods

*Animals:* Outbred Wistar male rats (Charles River) of an initial body weight of 150 ± 20 g were used throughout the experiments. Animals were subdivided in two groups (control and chronic smoker rats), kept under conventional conditions and weighed monthly. Groups of six rats each were lodged for 2 h, daily for 4 months in a smoke chamber as described previously,[9] during which animals breathed air containing the smoke of two cigarettes without filter (Camel). This particular brand was chosen only because it is an internationally known brand of cigarette. Immediately after the last smoking session and 8 and 24 h thereafter, animals received an intramuscular overdose of Nembutal (Serva) and blood was thereafter withdrawn from the femoral vein with an insulin-type syringe containing 10 IU/ml of heparin (Liquemin, Roche); 0.2 ml was used for the determination of carboxyhaemoglobin (COHb) and the remainder was centrifuged at 800 × *g* for 10 min. Plasma was stored at −80°C until the determination of cytokines. Afterwards 10 ml of sterile Hanks' balanced solution at a pH of 7.4, containing 5 IU/ml heparin, were injected into the peritoneal cavity, the abdomen was gently massaged and about 8 ± 1 ml were aspirated in sterile conditions for the collection of peritoneal M$\phi$ present in peritoneal fluid (PF). Bronchoalveolar lavage (BAL) was performed in the rats through a catheter inserted into the rat trachea with heparinized (0.1 IU/ml) sterile Hanks' solution. Five washes of 5 ml each were carried out in rapid succession and the lavage fluids were pooled. Recovery was about 90%.

Control animals (air-sham exposed rats) were subjected to the same procedure except that the chamber was insufflated with air only.

*Cell isolation and culture:* The recovered BAL and PF were centrifuged at 300 × *g* for 10 min at 4°C; supernatants were collected and stored at −80°C; cells were washed twice in cold RPMI 1640 medium (Gibco) and resuspended in a small volume of the same medium. After counting in a Bürker chamber, cells were diluted to the desired final concentration ($10^6$ viable cells/ml) with LPS-free RPMI 1640 containing 10% of heat-inactivated (56°C for 60 min) foetal calf serum (FCS), 2 mM L-glutamine, 100 U/ml penicillin G and 100 μg/ml streptomycin. The sedimented cells were proved to be macrophages (about 80% in the smokers) by nonspecific esterase staining. Separated M$\phi$ were tested for viability by blue exclusion method and more than 95% were found to be viable. Macrophages were cultured at a final volume of 0.2 ml per well in 96-well culture plates (Costar) and incubated for 24 h at 37°C in a humidified atmosphere (95% air: 5% $CO_2$). This period of time was chosen after preliminary experiments had shown that the highest TNF-α concentration was obtained after 24 h.[9] Plates were then centrifuged at 300 × *g* for 10 min at 4°C, supernatants were collected and stored at −80°C until TNF and IFN determinations. To verify cell responsiveness other lots of M$\phi$ suspension were challenged with 1 μg/ml bacterial lipopolysaccharide (LPS) from *Escherichia coli* (Sigma) and treated as above.

Proteins were measured directly in the wells using the method of Lowry *et al.*,[10] after two washings with saline and dissolving sedimented cells with 0.1 ml of 0.1 N NaOH for 12 h at room temperature. All media used were proved negative for endotoxin contamination by using the Limulus amoebocyte lysate gel test. Because the exposure of macrophages to endotoxin during the collection procedure cannot be absolutely excluded, supernatants of either BAL or PF were tested for endotoxin contamination and proved to be negative.

*COHb levels:* Blood COHb levels were estimated as an indicator for the biological effects of cigarette smoking. In order to check whether the smoke exposure was constant for all animals, the change of COHb in the blood of rats before and immediately after the smoking session was measured by a spectrophotometric method.[11]

*Cytokine determination:* Supernatants were titrated for antiviral activity (AA) with a microplaque reduction assay[12] using L929 murine cells and vesicular stomatitis virus (VSV, Indiana strain) as a challenge virus. Cell monolayers were infected with 50 plaque-forming units of VSV and all samples were tested twice in quadruplicate. Titrations were always performed employing the international reference preparation for murine IFN. Titres are reported as international units per ml (IU/ml).

TNF activity was determined by the method of Ruff and Gifford[13] with some modifications. Briefly, L929 cells were seeded at a density of 4 × $10^4$ cells per well in 96-well culture dishes in 100 μl of Eagle's MEM to which 10% FCS and antibiotics had been added. After 4 h of incubation at 37°C in a humidified atmosphere (95% air : 5% $CO_2$), two-fold serial dilutions of the samples in 2 μg/ml of actinomycin D (Serva), were prepared in separate 96-well culture dishes. Then 100 μl of each dilution was transferred into the corresponding well and plates, and further incubated for 18 h at 37°C in the humidified atmosphere. Supernatants were then removed and cells stained with 0.1% crystal violet. After drying, 100 μl of 33% acetic acid was added to each well to dissolve the dye. Plates were finally read at 540 nm on a Titertek Multiskan microElisa reader. Units of TNF activity were defined as the reciprocal of the dilution causing 50% of maximum cytotoxicity.

Human recombinant TNF-α (Biogen BASF/Knoll) with a specific activity of $1.5 \times 10^7$ units/mg proteins was used as internal standard. TNF-α activity was calculated as units/$10^6$ cells.

Because some subclones of L929 cells have been shown to be sensitive to either other cytokines or cytotoxic agents, in order to compare TNF-α biological activity with the TNF-α antigen really present in our samples, we performed some determinations using both biological assay and a commercial enzyme-linked immunosorbent assay (TNF–ALPHA–EASIA, Medgenix Diagnostics, Belgium), which showed no cross-reaction with other cytokines such as IFN and interleukins.

*Other chemical and enzymatic determinations:* Superoxide anion generation by alveolar Mϕ was measured as the superoxide dismutase-inhibitable reduction of ferricytochrome *c* in presence and absence of zymosan.[14] Results were expressed as nM/$10^5$ cells. Lactate dehydrogenase and β-glucuronidase were measured in the supernatants of BAL and PF by colorimetric kits (Sigma) and the results were reported as U/ml.

*Statistical analysis:* Evaluation of experimental data was performed using two-tailed Student's *t*-test with $p < 0.05$ as the minimal level of significance.

### Results

Body weight measured monthly during the 4 months of experiments was each time significantly lower ($p < 0.001$) in the smoker group than in air-sham exposed rats (Fig. 1).

After the last smoking session the blood COHb concentration in smoker rats was 8.5 ± 1.2% (Table 1). The total recovery of alveolar Mϕ from BAL remained practically constant in both smoker and control rats. In contrast, PMN in the BAL of smoker rats significantly increased in respect to air-sham exposed rats and, at the same time, peritoneal Mϕ in the PF decreased (Table 1).

When alveolar Mϕ were collected immediately after the last smoking session (0 h) and 8 and 24 h thereafter and were incubated for 20 min at 37°C in presence and absence of zymosan, superoxide anion generation increased significantly in respect to

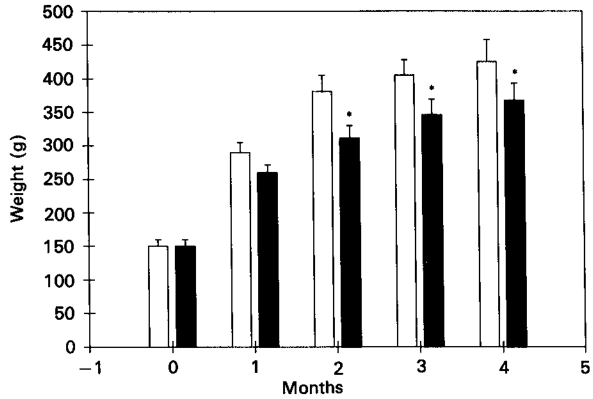

FIG. 1. Body weight expressed as mean ± S.D. measured monthly in both chronic smoker (full columns) and control (empty columns) rats. The values at 0 time represent the weight of the rats before the experiment. *$p < 0.001$ in respect to control values ($n = 20$).

control values, but remained unchanged after incubation of peritoneal Mϕ (Table 2).

Enzymatic analyses of BAL of smokers revealed a significant increase of β-glucuronidase and a decrease of lactate dehydrogenase, but both remained practically unvaried in the PF (Table 3).

As the results were consistent with a state of metabolic activation of the alveolar Mϕ population recovered from chronic smoke-exposed rats, we investigated whether these cells release TNF-α. Figure 2 (bottom panel) shows that alveolar Mϕ collected from smoker rats immediately after the last smoking session (0 h) and 8 and 24 h thereafter and incubated for 24 h at 37°C in a humidified atmosphere, spontaneously released 77 ± 28, 80 ± 23 and 84 ± 34 units of TNF-α per $10^6$ cells respectively, these values being significantly higher than those obtained after incubation of alveolar Mϕ collected from sham-exposed control rats. Moreover alveolar Mϕ obtained from BAL of both smoker and control rats challenged with 1 μg/ml of LPS and incubated as above, released much more TNF-α, but in such cases, TNF-α production by alveolar Mϕ of the smoker group was significantly decreased to about one half of that of the control group (Fig. 2, top panel). Immunoenzymatic determinations on samples, the biological activity of which is reported in Fig. 2, gave practically the same TNF-α units per $10^6$ cells (data not shown).

**Table 1.** HbCO in blood and concentration of total cells collected from bronchoalveolar fluid and peritoneal fluid of both control and smoker rats

| Parameter | Controls | Smokers |
|---|---|---|
| HbCO (%) | −1 ± 1.1 | 8.5 ± 1.2 |
| Alveolar Mϕ (×$10^6$) | 1.45 ± 0.5 | 1.67 ± 0.41 |
| Alveolar PMN (×$10^5$) | 0.17 ± 0.09 | 0.33 ± 0.14** |
| Peritoneal Mϕ (×$10^6$) | 9.23 ± 2.65 | 6.72 ± 2.5* |

*$p < 0.05$ and **$p < 0.01$ in respect to control values ($n = 18$).

**Table 2.** Superoxide anion (nM/$10^5$ cells) produced by alveolar and peritoneal macrophages, collected from both smoker and control rats 0, 8 and 24 h after the last smoking session

| Time after the smoking session (h) | Alveolar Mϕ | | Peritoneal Mϕ | |
|---|---|---|---|---|
| | Controls | Smokers | Controls | Smokers |
| 0 | 5.3 ± 4.5 | 12.4 ± 2* | 5.3 ± 2.9 | 6.7 ± 2.4 |
| 8 | 4.4 ± 0.8 | 31.8 ± 12.7* | 6.2 ± 3.1 | 7.5 ± 2.5 |
| 24 | 5.7 ± 1.6 | 15.2 ± 4.4* | 8.2 ± 3.3 | 11.5 ± 3 |
| Total mean | 5.2 ± 2.9 | 18.6 ± 9.9** | 6.6 ± 3 | 8.6 ± 3.2 |

*$p < 0.05$ ($n = 6$) and **$p < 0.001$ ($n = 18$) in respect to control values.

**Table 3.** Lactate dehydrogenase and β-glucuronidase measured in the supernatant of bronchoalvedar fluid and peritoneal fluid, both collected from chronic smoker and control rats 0, 8 and 24 h after the last smoker session

| Time after the smoking session (h) | Supernatant bronchoalveolar fluid | | | | Supernatant peritoneal fluid | | | |
|---|---|---|---|---|---|---|---|---|
| | Lactate dehydrogenase (U/ml) | | β-glucuronidase (U/ml) | | Lactate dehydrogenase (U/ml) | | β-glucuronidase (U/ml) | |
| | Controls | Smokers | Controls | Smokers | Controls | Smokers | Controls | Smokers |
| 0 | 83 ± 14 | 76 ± 33 | 1 ± 0.6 | 2.9 ± 0.9** | 93 ± 23 | 161 ± 106 | 1.8 ± 1.7 | 7.7 ± 3.9* |
| 8 | 97 ± 8 | 63 ± 10* | 1.4 ± 0.3 | 2.6 ± 0.8* | 169 ± 79 | 64 ± 54 | 4.7 ± 1.4 | 6.3 ± 3.7 |
| 24 | 72 ± 20 | 59 ± 13 | 0.7 ± 0.16 | 3.1 ± 0.7** | 99 ± 19 | 39 ± 14* | 2 ± 2 | 6.5 ± 3.8 |
| Total mean | 85 ± 17 | 67 ± 23* | 1.1 ± 0.5 | 2.8 ± 0.8*** | 118 ± 56 | 102 ± 94 | 2.7 ± 2 | 7 ± 3.6 |

*$p < 0.5$ ($n = 6$), **$p < 0.01$ ($n = 6$) and ***$p < 0.001$ ($n = 18$), in respect to control values.

Alveolar Mϕ collected from both smoker and control rats were unable to release any antiviral activity and, either TNF-α, or IFN were also absent in the supernatants of BAL and PF and in plasma (data not shown). Instead peritoneal Mϕ of both smoker and control rats released, either spontaneously, or after LPS induction, little and extremely variable amounts of TNF-α and IFN (Table 4). We attempted to correlate individual ratios of TNF-α released by alveolar Mϕ compared with TNF-α released by peritoneal Mϕ of both control and smoker rats. As shown in Table 5 ratios obtained from smokers were always higher than those obtained from controls although extremely variable.

### Discussion

Some biological effects of chronic smoke in rats have been evaluated. During the 4 months of the smoking period the body-weight increments were always lower than those of control rats. Although the cigarette smoking effects in lowering the body-weight are well known,[15,16] little attention has been devoted to studying the effect of chronic smoke on the metabolic rate in rats where sympathoadrenal activation by nicotine appears to be primarily responsible for the metabolic effects of smoking.[17]

Smoking rats were lodged in the smoking chamber daily for 2 h, during which time they breathed the smoke of two cigarettes. Evaluation of COHb blood levels, immediately after the last smoking session, gave values of about 8.5% which compared well with those observed in guinea pig and in human cigarette smokers.[18,19] The total number of alveolar Mϕ collected from BAL of chronic smoker rats was unchanged compared with that of control rats or acute smoker rats,[9] but PMN increased significantly in the BAL of smoker rats. Previous work by Costabel and Guzman[20] and others[2,21,22] clearly shows a constant increase of PMN in the BAL after smoking, probably due to the action of chemotactic factors[23,24] produced by both alveolar and pulmonary cells.

The major aim of the investigation reported here was to determine whether resident alveolar Mϕ of chronic smoker rats produced TNF-α. The present results clearly demonstrated that alveolar Mϕ collected immediately after (0 h) and 8 and 24 h following the last smoking session and incubated for 24 h at 37°C spontaneously released significant and constant amounts of TNF-α. Immunoenzymatic analyses on these samples gave about the same values, thus assuring the identity of the cytokine. We are unable to assess which substance stimulated the release of TNF-α and at least IFN-γ[25] can be excluded, because we were unable to detect any antiviral activity in our samples. TNF-α and IFN were also absent in the supernatants of BAL and PL, but this is probably due to the dilution and short time of contact of these fluids with the cells.

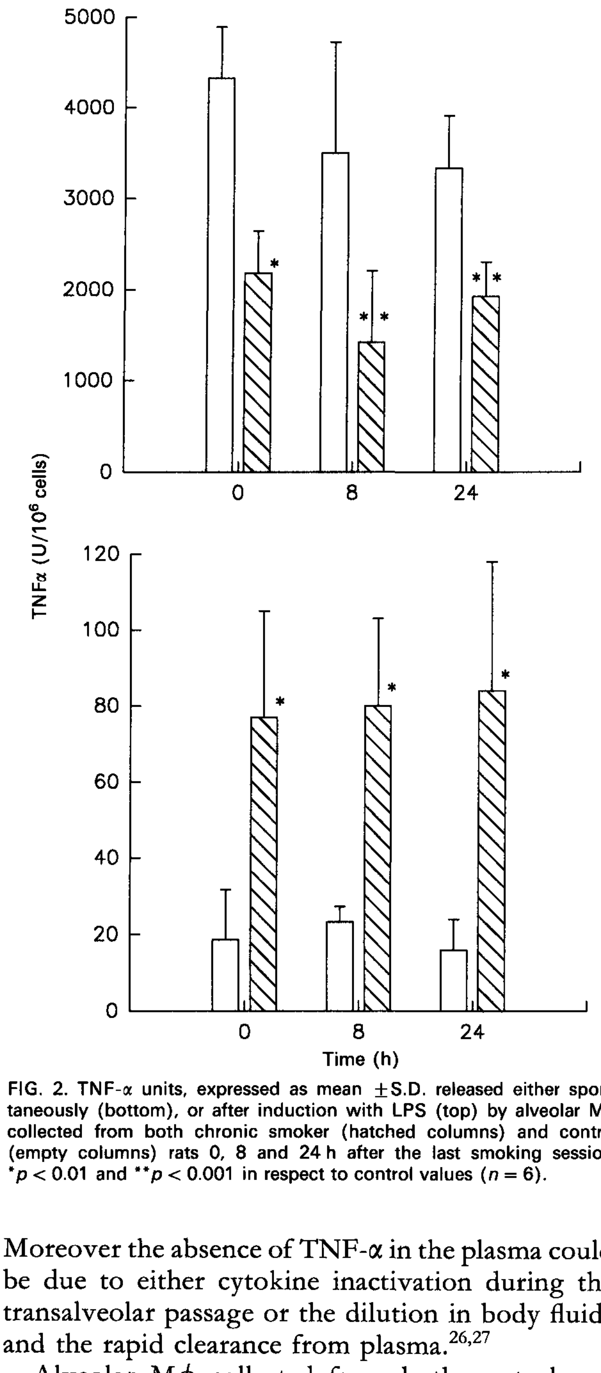

FIG. 2. TNF-α units, expressed as mean ±S.D. released either spontaneously (bottom), or after induction with LPS (top) by alveolar Mϕ collected from both chronic smoker (hatched columns) and control (empty columns) rats 0, 8 and 24 h after the last smoking session. *$p < 0.01$ and **$p < 0.001$ in respect to control values ($n = 6$).

Moreover the absence of TNF-α in the plasma could be due to either cytokine inactivation during the transalveolar passage or the dilution in body fluids and the rapid clearance from plasma.[26,27]

Alveolar Mϕ collected from both control and smoker rats were extremely sensitive to low LPS concentrations but, in the last case, TNF-α release was about one-half of that observed in the control group, in agreement with results of others obtained in the mouse.[28] Peritoneal Mϕ of the control and smoker group, which represent a resident population without any direct contact with air pollutants and smoke products, released small amounts of TNF-α and IFN either spontaneously or after challenge with LPS. This is also clarified by the analysis of individual ratios of alveolar TNF-α versus peritoneal TNF-α released various time after the last smoking session evaluated in both control and smoker rats. The high variability of the ratios is probably due to the scarce sensitivity of our bioassay especially when the units of TNF-α were below 5. Taken together, the results suggest that peritoneal Mϕ were in a resting state and alveolar Mϕ were primed, based on the fact that only primed Mϕ are able to release massive amounts of TNF-α after stimulation with LPS.[29,30] Moreover it is known[29] that at least two pathways govern the release of TNF-α, one is LPS specific and the other phagocytic trigger specific, suggesting that the priming effect for the TNF-α release is variable and probably related to a particular triggering stimulus. The activated state of alveolar Mϕ of chronic smoker rats, due to either smoke products and pollutants,[31,32] or a self-priming effect of TNF-α[33–35] is also suggested by the significant increase of superoxide anion generation and of β-glucuronidase. At the same time lactate dehydrogenase in the BAL of smoker rats, which represents a cytoplasmic marker employed as a measure of the cell lysis, remained unchanged or only slightly

**Table 5.** Individual ratios expressed as mean ± S.D. of TNF-α obtained from alveolar *vs.* peritoneal macrophages in both control and smoker rats 0, 8 and 24 h after the last smoking session

| Time after the last smoking session (h) | Ratios | |
|---|---|---|
| | Controls | Smokers |
| 0 | 0.6 ± 0.3 | 20.2 ± 35.5 |
| 8 | 1.2 ± 0.3 | 4.6 ± 1.5 |
| 24 | 5.7 ± 3.1 | 76 ± 46 |

**Table 4.** TNF-α and IFN (IU/$10^6$ cells) expressed as mean ±S.D. produced by peritoneal macrophages collected from both chronic smoker and control rats 0, 8 and 24 h after the last smoking session and incubated for 24 h at 37°C

| Time after the smoking session (h) | Peritoneal macrophages | | | | | | | |
|---|---|---|---|---|---|---|---|---|
| | Controls | | Controls ± LPS | | Smokers | | Smokers ± LPS | |
| | TNF | IFN | TNF | IFN | TNF | IFN | TNF | IFN |
| 0 | 30 ± 11 | 7.4 ± 8.5 | 36 ± 12 | 11 ± 9 | 12 ± 7 | 19 ± 17 | 20 ± 6 | 20 ± 18 |
| 8 | 29 ± 6 | 2.6 ± 5.8 | 49 ± 6 | 8±8 | 19 ± 10 | 15 ± 11 | 35 ± 30 | 16 ± 10 |
| 24 | 25 ± 18 | 26 ± 29 | 25 ± 18 | 29 ± 25 | 2 ± 4 | 1 ± 2 | 7 ± 3 | 2 ± 4 |
| Total mean | 23 ± 13 | 11 ± 18 | 37 ± 15 | 16 ± 17 | 10 ± 9 | 11 ± 14 | 20 ± 18 | 13 ± 15 |

decreased compared with the control group. This suggests that the increase of the $\beta$-glucuronidase in the BAL was not due to a lytic effect on the cells, but to a secretagogue effect on alveolar M$\phi$ and PMN probably exerted by smoke products and/or TNF-$\alpha$ itself.[35]

### References

1. Roberts DL. Natural tobacco flavor. *Recent Adv Tobacco Sci* 1988; **14**: 49–81.
2. Robbins RA, Thompson AB, Koyama S, Metcalf JP, Rickard KA, Spurzem JR, Rennard SI. Cigarette smoking and neutrophil migration. *J Immunol Res* 1990; **2**: 178–188.
3. Kelley J. Cytokines of the lung. *Am Rev Respir Dis* 1990; **141**: 765–788.
4. Elias JA, Freundlich B, Kern JA, Rosenbloom J. Cytokine networks in the regulation of inflammation and fibrosis in the lung. *Chest* 1990; **97**: 1439–1445.
5. Bonta IL, Ben-Efraim S, Mozes T, Flieren MWJA. Tumor necrosis factor in inflammation relation to other mediators and to macrophage antitumour defence. *Pharm Res* 1991; **24**: 115–129.
6. Adams DO, Hamilton FA. Phagocytic cells: cytotoxic activities of macrophages. In: Gallin JI, Goldstein M, Snyderman R eds. *Inflammation: basic principles and clinical correlates*. New York: Raven Press, 1988; 471–492.
7. Vilcek J, Lee TH. Tumor Necrosis factor. New insights into the molecular mechanisms of its multiple actions. *J Biol Chem* 1991; **266**: 7313–7316.
8. Camussi G, Alano E, Tetta C, Bussolino F. The molecular action of tumor necrosis factor-α. *Eur J Biochem* 1991; **202**: 3–14.
9. Pessina GP, Paulesu L, Corradeschi F *et al.* Effects of acute cigarette smoke exposure on macrophage kinetics and release of tumour necrosis factor in rats. *Mediators of Inflammation* 1993; **2**: 119–122.
10. Lowry OH, Rosebrough NJ, Farr AL, Randall RJ. Protein measurement with the folin phenol reagent. *J Biol Chem* 1951; **193**: 265–275.
11. Kita H, Kubota T, Muroya H. A rapid spectrophotometric determination of carboxyhemoglobin in blood. *Med Biol* 1973; **87**: 305.
12. Langford MP, Weigent DA, Stanton GJ, Baron S. Virus plaque reduction assay for interferon: microplaque and regular macroplaque reduction assays. In: Pestka S, ed. *Methods of Enzymology*. New York: Academic Press, Inc., 1981; 339–346.
13. Ruff MR, Gifford GE. Tumor necrosis factor. In: Pick E, ed. *Lymphokines*. New York: Academic Press, 1981; 235–241.
14. Bellavite P, Dri P, Berton G, Zabucchi G. Un nuovo test di funzionalità fagocitaria basato sulla misura della produzione di anione superossido (2*). 1. Principi generali ed esecuzione. *Lab J Res Lab Med* 1980; **VII**: 67–76.
15. Kitchen JMW. On the value to man of the so-called divinely beneficient gift, tobacco. *Med Rec* 1989; **35**: 459–460.
16. Klesges RC, Klesges LM. Cigarette smoking as a dieting strategy in a university population. *Int J Eat Dis* 1988; **7**: 413–419.
17. Perkins KA. Metabolic effects of cigarette smoking. *J Appl Physiol* 1992; **72**: 401–409.
18. Churg A, Tron V, Wright JL. Effects of cigarette smoke exposure on retention of asbestos fibers in various morphologic compartments of the guinea pig lung. *Am J Pathol* 1987; **129**: 385–393.
19. Prignot J. Quantification and chemical markers of tobacco exposure. *Eur J Respir Dis* 1987; **70**: 1–7.
20. Costabel U, Guzman J. Effect of smoking on bronchoalveolar lavage constituents. *Eur Resp J* 1992; **5**: 776–779.
21. MacNee W, Wiggs B, Belzberg AS, Hogg JC. The effect of cigarette smoking on neutrophil kinetics in human lungs. *N Engl J Med* 1989; **321**: 924–928.
22. Burke WMJ, Roberts CM, Bryant DH *et al.* Smoking-induced changes in epithelial lining fluid volume, cell density and protein. *Eur Resp J* 1992; **5**: 780–784.
23. Kunkel SL, Standiford T, Kasahara K, Strieter RM. Interleukin-8 (IL-8): the major neutrophil chemotactic factor in the lung. *Exp Lung Res* 1991; **17**: 17–23.
24. Issekutz AC, Morzycki W, Sadowska J. Rabbit alveolar macrophages stimulated with endotoxin and lung fragments from endotoxemic rabbits produce a leukocyte infiltration-inducing factor that lacks IL-1, TNF alfa*, or chemotactic activity. *Exp Lung Res* 1991; **17**: 803–819.
25. Wilson BMG, Severn A, Rapson NT, Chana J, Hopkins P. A convenient human whole blood culture system for studying the regulation of tumour necrosis factor release by bacterial lipopolysaccharide. *J Immunol Methods* 1991; **139**: 233–240.
26. Bocci V, Pacini A, Pessina GP, Maioli E, Naldini A. Studies on tumor necrosis factor (TNF). 1. Pharmacokinetics of human recombinant TNF in rabbits and monkeys after intravenous administration. *Gen Pharm* 1987; **18(4)**: 343–346.
27. Pessina GP, Pacini A, Bocci V, Maioli E, Naldini A. Studies on tumor necrosis factor (TNF). II. Metabolic fate and distribution of human recombinant TNF. *Lymphokine Res* 1987; **6**: 35–44.
28. Higashimoto Y, Shimada Y, Fukuchi Y *et al.* Inhibition of mouse alveolar macrophage production of tumor necrosis factor alpha by acute *in vivo* and *in vitro* exposure to tobacco smoke. *Respiration* 1992; **59**: 77–80.
29. Stein M, Gordon S. Regulation of tumor necrosis factor (TNF) release by murine peritoneal macrophages: role of cell stimulation and specific phagocytic plasma membrane receptors. *Eur J Immunol* 1991; **21**: 431–437.
30. Mohr C, Gemsa D, Graebner C *et al.* Systemic macrophage stimulation in rats with silicosis: enhanced release of tumor necrosis factor-α from alveolar and peritoneal macrophages. *Am J Respir Cell Mol Biol* 1991; **5**: 395–402.
31. Drath DB, Karnovsky ML, Huber GL. Tobacco smoke. Effects on pulmonary host defense. *Inflammation* 1979; **3**: 281–288.
32. Anderson R. Assessment of the roles of vitamin C, vitamin E, and β-carotene in the modulation of oxidant stress mediated by cigarette smoke-activated phagocytes 1–3 (sup). *Am J Clin Nutr* 1991; **53**: 358S–361S.
33. Berkow RL, Wang D, Larrick JW, Dodson RW, Howard TH. Enhancement of neutrophil superoxide production by preincubation with recombinant human tumor necrosis factor. *J Immunol* 1987; **139**: 3783–3791.
34. Bajaj MS, Kew RR, Webster RO, Hyers TM. Priming of human neutrophil functions by tumor necrosis factor: Enhancement of superoxide anion generation, degranulation, and chemotaxis to chemoattractants C5a and F-Met-Leu-Phe. *Inflammation* 1992; **16**: 241–250.
35. Klebanoff SJ, Vadas MA, Harlan JM *et al.* Stimulation of neutrophils by tumor necrosis factor. *J Immunol* 1986; **136**: 4220–4225.

ACKNOWLEDGEMENTS. This work was supported by contracts no. 92.00024.PF41 and 93.00594.PF41, Project FATMA, Subproject SP2, CNR, Rome and in part by a contribution of MURST.

**Received 30 July 1993; accepted in revised form 20 September 1993**
